# Targeted Genomic Integration of a Selectable Floxed Dual Fluorescence Reporter in Human Embryonic Stem Cells

**DOI:** 10.1371/journal.pone.0046971

**Published:** 2012-10-10

**Authors:** Jay A. Gantz, Nathan J. Palpant, Robert E. Welikson, Stephen D. Hauschka, Charles E. Murry, Michael A. Laflamme

**Affiliations:** 1 Department of Bioengineering, Center for Cardiovascular Biology, Institute for Stem Cell and Regenerative Medicine, University of Washington, Seattle, Washington, United States of America; 2 Department of Pathology, Center for Cardiovascular Biology, Institute for Stem Cell and Regenerative Medicine, University of Washington, Seattle, Washington, United States of America; 3 Department of Biochemistry, Center for Cardiovascular Biology, Institute for Stem Cell and Regenerative Medicine, University of Washington, Seattle, Washington, United States of America; 4 Department of Medicine/Cardiology, Center for Cardiovascular Biology, Institute for Stem Cell and Regenerative Medicine, University of Washington, Seattle, Washington, United States of America; Stanford University School of Medicine, United States of America

## Abstract

The differentiation of pluripotent stem cells involves transition through a series of specific cell states. To understand these cell fate decisions, the field needs improved genetic tools for the labeling, lineage tracing and selection of specific cell types from heterogeneous differentiating populations, particularly in the human embryonic stem cell (hESC) system. We used zinc finger nuclease technology to stably insert a unique, selectable, floxed dual-fluorescence reporter transgene into the AAVS1 locus of RUES2 hESCs. This “stoplight” transgene, mTmG-2a-Puro, strongly expresses membrane-localized tdTomato red fluorescent protein until Cre-dependent recombination causes a switch to expression of membrane-localized enhanced green fluorescent protein (eGFP) and puromycin resistance. First, to validate this system in undifferentiated cells, we transduced transgenic hESCs with a lentiviral vector driving constitutive expression of Cre and observed the expected phenotypic switch. Next, to demonstrate its utility in lineage-specific selection, we transduced differentiated cultures with a lentiviral vector in which the striated muscle-specific CK7 promoter drives Cre expression. This yielded near-homogenous populations of eGFP^+^ hESC-derived cardiomyocytes. The mTmg-2a-Puro hESC line described here represents a useful new tool for both in vitro fate mapping studies and the selection of useful differentiated cell types.

## Introduction

The successful development of safe and efficacious stem cell-based therapies will require an improved understanding of the lineage relationships between stem cells and their differentiating progeny, as well as the detailed molecular properties of cells in the initial, transitional and final differentiated states. In model organisms (e.g. Xenopus, Drosophila, mouse etc.), these issues have often been addressed by fate mapping studies using elegant genetic labeling approaches, in particular, the Cre-lox system. Currently, there are over 500 genetically modified mouse lines expressing Cre recombinase under the control of various promoter elements, as well as a large cohort of transgenic lines with Cre-responsive reporter elements [1].

While human embryonic stem cells (hESCs) have become a valuable in vitro model of human development and a potential source for cell-based therapies, Cre-lox mediated fate mapping has not been widely applied to hESCs. In this report, zinc finger nuclease (ZFN)-mediated genetic engineering was used to generate stable human ESC line expressing a selectable floxed dual fluorescence reporter element. The random integration of transgenes is susceptible to silencing, positional effects and off-target effects on cellular function, so we instead targeted our reporter to the so-called “safe harbor” AAVS1 locus, which affords stable transgene expression in a wide variety of cell types including hESCs [2]. The reporter element couples a fluorescence color switch with puromycin resistance to enable isolation of nearly homogeneous cellular subtypes from heterogeneous populations after Cre-mediated recombination. Here, we demonstrate the efficacy of this system for the purification of differentiated cardiomyocytes from hESCs, but it should be widely applicable to the selection of diverse cell lineages when combined with appropriate cell type-specific regulatory cassettes driving Cre.

## Methods

### Zinc Finger Nuclease Co-expression Plasmid

**Figure 1:**
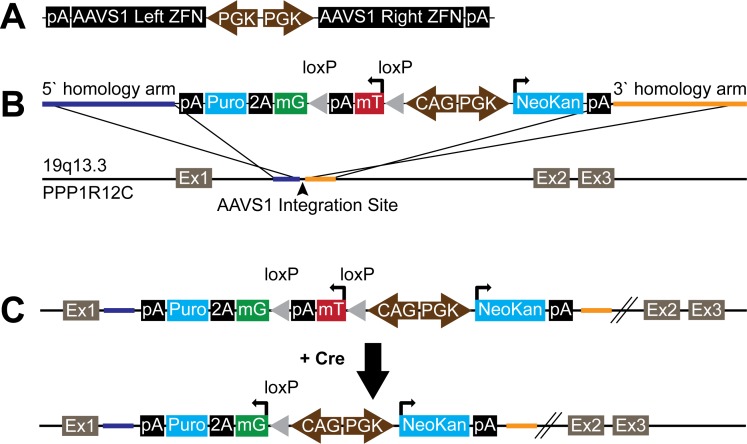
Transgene plasmid construction. (**A**) pAAVS1ZFN vector diagram showing two reverse orientation human PGK promoters driving the left and right AAVS1 zinc finger nucleases (ZFNs). (**B**) pZDonor-mTmG-2a-Puro vector diagram and the AAVS1 genomic integration site on chromosome 19 in the first intron of the PPP1R12C gene. The PGKNeoKan cassette enables selection of cells with stable transgene integration and the CAG-mTmG-2a-Puro selectable “stoplight” cassette allows for visual and antibiotic selection of cells that have been recombined by Cre recombinase. Yellow and blue colored bars represent ∼800 bp stretches of sequence homologous to either side of the AAVS1 target locus. (**C**) In coordination with the AAVS1 ZFNs, the mTmG-2a-Puro transgene was integrated into the AAVS1 site by homologous recombination. Cre mediated recombination of LoxP sites removes the tdTomato expression cassette and results in eGFP-2a-Puro expression. Abbreviations: pA  =  bovine growth hormone polyadenylation signal, mT  =  myristoylated tdTomato, mG  =  myristoylated eGFP, NeoKan  =  neomycin/kanamicin resistance, Puro  =  puromycin resistance, loxP  =  Cre recombinase locus of chromosomal crossover, Ex  =  exon.

To target the donor cassette to the AAVS1 human genomic locus, we generated a single plasmid system in which expression of the left and right AAVS1 ZFNs are both driven by independent, constitutively active human PGK promoters (pAAVS1ZFN; see **Figure 1A**). The human PGK promoter was kindly provided by Dr. Mark Mercola (Sanford Burnham Institute for Medical Research). Sequences for the AAVS1 right and left ZFNs were generated by human codon optimization of the amino acid sequences reported by Hockemeyer et al [2]. The nucleotide sequences for the right and left ZFNs driven by the PGK promoter were then synthesized de novo as separate plasmids by Genscript. These PGK-ZFN cassettes were then cloned together in opposing orientation into a single expression plasmid, which was then sequence-verified. Of note, we initially compared outcomes using two different versions of this expression plasmid in which the PGK-ZFN cassettes were oriented in either the same or opposite direction. Interestingly, we only observed successful targeting and the emergence of fluorescent hESCs when using the plasmid with the two cassettes in the opposing orientation.

### AAVS1 Targeting Vector

The AAVS1 donor vector, pZDonor mTmG-2a-Puro was generated as follows. A plasmid containing the floxed non-selectable dual fluorescence reporter (mTmG) by Muzumdar et al. [3] was acquired commercially (Addgene, 17787). A targeting vector for the human AAVS1 locus (19q13.3-qter), pZDonor, was provided with the AAVS1 CompoZr Targeted Integration kit (Sigma, Z3027-100UG). The mTmG plasmid was restriction digested with EcoRV, EcoRI, and SacI to isolate the 5.6 kb fragment containing the CAG promoter and mTmG expression cassette. The pZDonor vector was restriction digested with EcoRV and PmeI into which the 5.6 kb mTmG expression cassette was ligated. The E2a element containing a unique XhoI site at the 3′ end was inserted using PCR-cloning distal to the mTmG expression cassette. The puromycin resistance cassette was inserted using Infusion-HD (Clontech) into the XhoI site. A cassette encoding for PGK-driven expression of neomycin/kanamycin resistance was cloned in the reverse orientation into a SalI site 5′ of the CAG mTmG-2a-Puro cassette. The resultant pZDonor mTmG-2a-Puro plasmid (**Figure 1B**) was then sequence verified.

**Figure 2:**
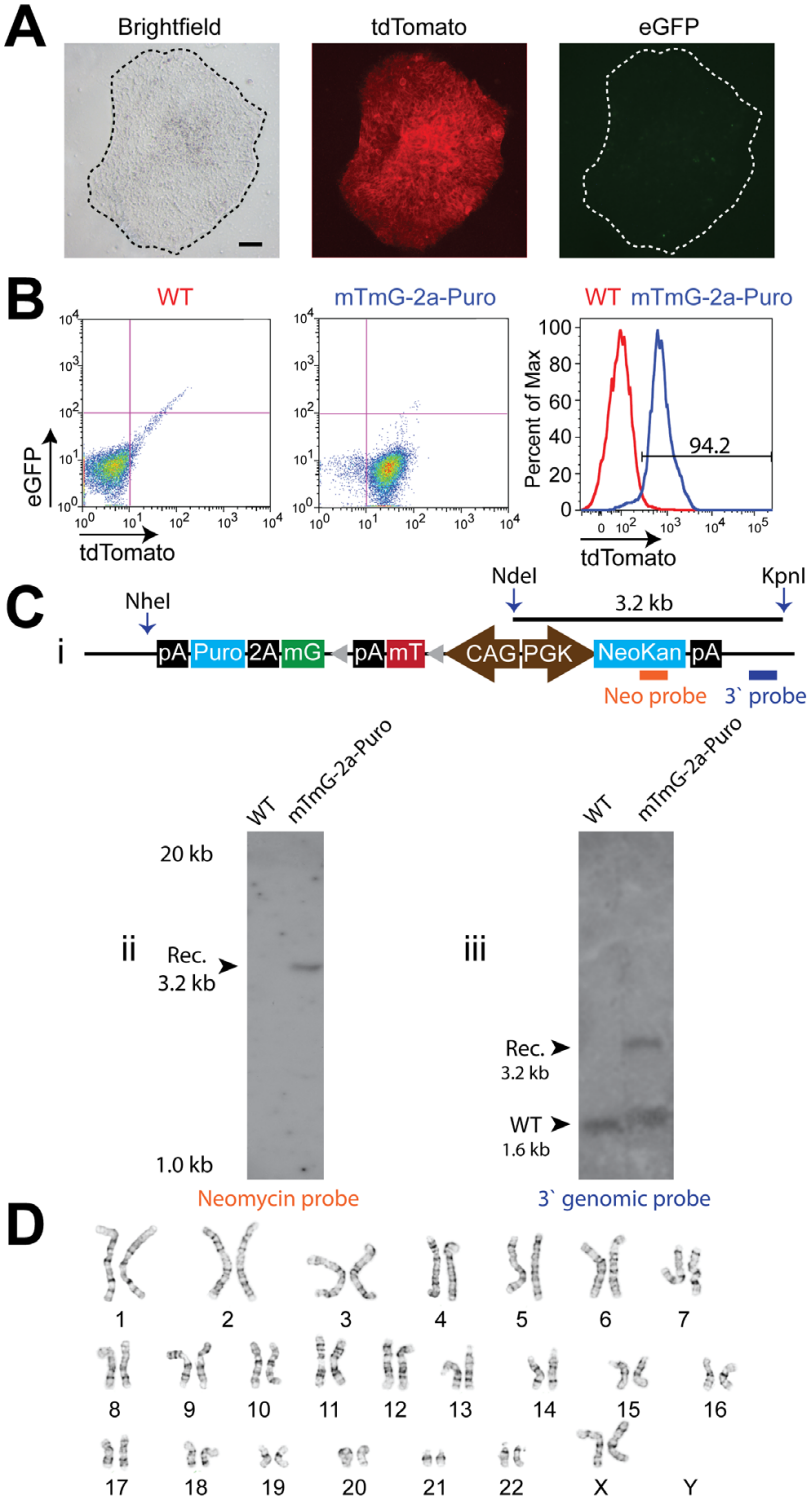
Generation of stable transgenic RUES2 mTmG-2a-Puro undifferentiated hESCs. Photomicrographs of an undifferentiated mTmG-2a-Puro RUES2 colony (**A**) under brightfield, tdTomato (red) and eGFP (green) fluorescent illumination. Note that the transgenic hESCs are tdTomato^+^ and eGFP^-^. Scale bar is 75 µm. (**B**) eGFP and tdTomato flow cytometry of wild type RUES2 hESCs (WT) and RUES2 mTmG-2a-Puro undifferentiated hESCs after G418 selection (mTmG-2a-Puro). G418 selection produces >94% purity of mTmG-2a-Puro cells as measured by flow cytometry for tdTomato fluorescent expression (see the panel on the right). (**C**) (i) Schematic showing the NheI, NdeI and KpnI cut sites within the transgene. The orange bar represents the binding site for the neomycin probe. The blue bar represents the binding site for the 3′ genomic probe. Non-locus-specific Southern blot detection of the neomycin transgene detects only a single copy of the mTmG-2a-Puro construct (ii), while Southern blot analysis of the AAVS1 locus shows heterozygous targeting of the mTmG-2a-Puro transgene to the AAVS1 locus (iii). (**D**) G-banded karyotyping of mTmG-2a-Puro RUES2 undifferentiated cells demonstrating a normal (46, XX) karyotype.

**Figure 3:**
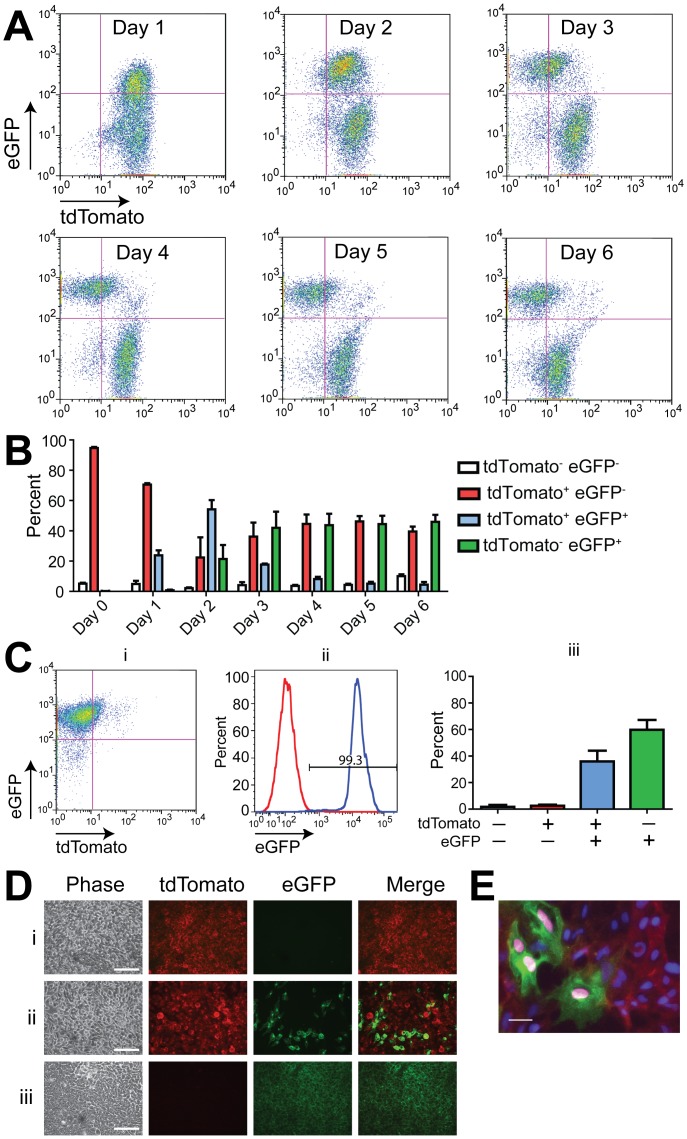
Cre expression mediates a fluorescence switch in undifferentiated mTmG-2a-Puro floxed reporter hESCs. (**A**) Flow cytometry showing changing numbers of tdTomato^+^ and eGFP^+^ RUES2 mTmG-2a-Puro cells at various timepoints after transduction with an EF1α-Cre lentivirus. (**B**) Quantitation of the fluorescence switch shown in panel A (n = 3 biological replicates). (**C**) Scatter plot of undifferentiated mTmG-2a-Puro cells treated with EF1α-Cre lentivirus and then selected with puromycin for 48 hours. Note that the resultant cultures were >99% eGFP^+^ by flow cytometry (i). Comparison of untreated (red) and EF1α-Cre treated, puromycin selected (blue) undifferentiated mTmG-2a-Puro cells (ii). Quantitation of flow cytometry data from three independent experiments in which cells were transduced with EF1α-Cre and puromycin selected (iii). (**D**) Phase contrast and fluorescent photomicrographs of undifferentiated mTmG-2a-Puro cells (i), mTmG-2a-Puro cells treated with EF1α-Cre lentivirus (ii), and mTmG-2a-Puro cells treated with EF1α-Cre and selected with puromycin (iii). Scale bars are 50 µm. Error bars represent +/− one standard error of the mean. (**E**) mTmG-2a-Puro cells transduced with EF1α-Cre lentivirus were immunostained with an anti-Cre recombinase antibody. Note that Cre (magenta) co-localizes only with eGFP^+^ cell nuclei. Scale bar is 10 µm.

**Figure 4:**
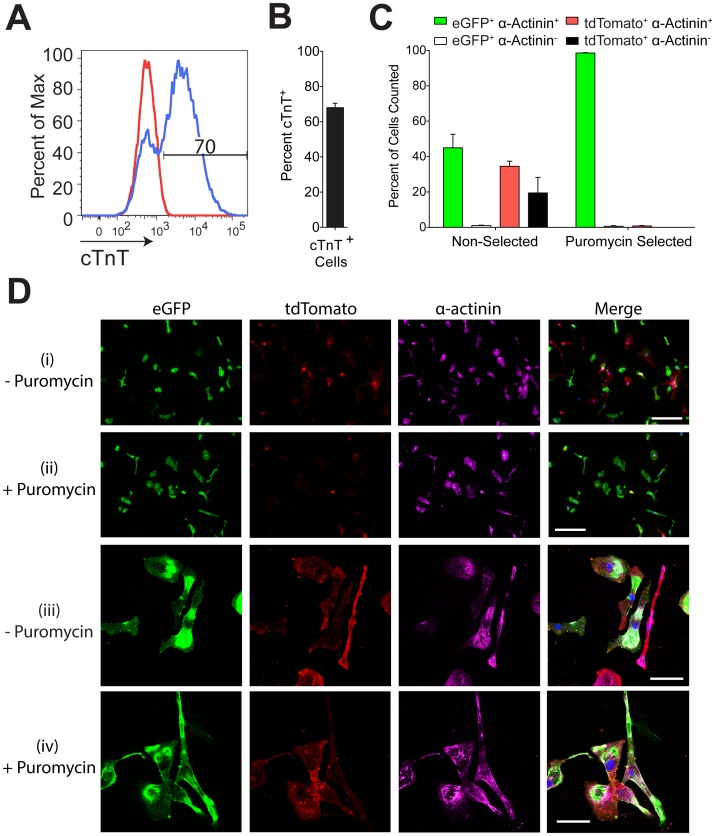
Using CK-7 Cre in combination with mTmG-2a-Puro cells enables cardiomyocyte purification. (**A**) Flow cytometry for cardiac troponin T (cTnT) indicating the percentage of cardiomyocytes present after mTmG-2a-Puro hESCs were subjected to a directed cardiac differentiation protocol. (**B**) Mean percentage of cTnT^+^ cells resulting from three independent differentiation runs. (**C**) The percentage of cells that immunostained for eGFP, tdTomato, and/or the cardiomyocyte marker α-actinin was determined before and after puromycin selection (n = 230–600 cells per condition for three independent differentiation runs). (**D**) Magnification at 10x (i and ii) and 60x (iii and iv) showing fluorescent photomicrographs of cardiac differentiated mTmG-2a-Puro cells before (i and iii) and after (ii and iv) puromycin selection. Scale bars are 200 µm in (i and ii) and 50 µm in (iii and iv). Error bars represent one standard error of the mean.

### Genomic Integration and Cell Culture

RUES2 hESCs were kindly provided by Dr. Ali H. Brivanlou of The Rockefeller University [4], [5]. RUES2 hESCs are listed on the NIH Human Embryonic Stem Cell Registry (Approval Number: NIHhESC-09-0013), and all experiments were approved by the University of Washington Embryonic Stem Cell Research Oversight (ESCRO) Committee. 5×10^5^ undifferentiated RUES2 hESCs were dispersed to single cells using Versene (Gibco) and electroporated with 5 µg of pAAVS1ZFN and 15 µg of pZDonor mTmG-2a-Puro (Lonza, Kit 1 (Cat#: VAPH-5012), program A-23). After electroporation, hESCs were plated onto Matrigel (BD Biosciences) coated plates in mouse embryonic fibroblast (MEF) conditioned media containing 5 ng/mL hbFGF (Peprotech) and 10 uM Y-27632 (Sigma). The cultures were maintained for 7 days and then selected with 75 µg/mL G418 (Invitrogen) in MEF conditioned media for 3 days. A polyclonal tdTomato^+^, G418-resistant RUES2 hESC population was expanded and analyzed for purity by flow cytometry. Karyotype analysis was performed by Cell Line Genetics (Madison, Wisconsin). Thereafter, RUES2 mTmG-2a-Puro hESCs showing proper targeting to the AAVS1 locus and a normal karyotype, were maintained under feeder-free conditions, using MEF-conditioned media supplemented with 5 ng/mL hbFGF [6]. These cells and plasmids are available for non-commercial distribution via a standard Uniform Biological Materials Transfer Agreement with our institution.

### Kinetics Studies

To determine the kinetics of the transition from tdTomato to eGFP expression, RUES2 mTmG-2a-Puro cultures were dispersed with Versene and transduced with a lentiviral vector in which expression of Cre recombinase is driven by the constitutive EF1α promoter. Cells were harvested and analyzed by flow cytometry at 24 hour intervals for up to 6 days following transduction. To test the capacity of our system to purify an eGFP^+^ sub-population after Cre-mediated recombination, transduced cultures were also subjected to selection with 1 µg/mL puromycin for 4 days and analyzed by flow cytometry. Flow cytometry was performed using a FACSCANTO 2 instrument (Beckton Dickinson, San Jose, CA) controlled by FACSDiva software (BD Biosciences). Cells were analyzed using a 488-nm argon laser plotting PE (585/42 filter) against FITC (530/30 filter). Instrument settings were adjusted to avoid spectral overlap. Data analysis was performed using FlowJo software (Tree Star, Ashland, Oregon).

### Southern Blot

15 µg of WT RUES2 and mTmG-2a-Puro RUES2 genomic DNA were digested with KpnI, NdeI, and NheI and electrophoresed on a 1% polyacrylamide gel. DNA was transferred onto a BioRad Zeta Probe membrane (Cat#: 162-0196), which was washed with 2x SSC, dried at 80°C in a hybridization oven for 2 hours, and then exposed for 1 hour to a pre-hybridization buffer (50% formamide, 0.12 M NaH2PO4, 0.25 M NaCl, 7% SDS, 1 mM EDTA and 0.2 mg/mL Salmon Sperm DNA). The hybridization probe for the 3′ genomic region of the AAVS1 locus was generated using the following primers: Sense: TGGGCGGAGGAATATGTCCCA; antisense: CACACCCCCATTTCCTGGAGC. The probe for the neomycin cassette was generated using the following primers: Sense: AGTCGATGAATCCAGAAAAGC; antisense: GCCGTGTTCCGGCTGTCAG. The probes were labeled with ^32^P dCTP (Amersham Megaprime DNA labeling system, RPN1607 with non-incorporated radiolabeled nucleotides removed by column purification (Illustra Microspin G-50 columns, GE Healthcare, Cat# 27-5330-01). Probe hybridization was performed overnight in hybridization buffer at 43°C. After 24 hours, the membrane was washed for 20 minutes with 2x SSC/0.1% SDS followed by 20 minutes in 0.1x SSC/0.1% SDS. The membrane was then exposed to autoradiographic film for 3 days. The WT AAVS1 band is expected at 1.6 kb whereas the targeted locus is expected to shift to 3.2 kb. Note that the neomycin probe is not locus-specific and indicates the number of genomic integration sites.

### Human ESC Cardiac Differentiation

The cardiac differentiation of RUES2 mTmG-2a-Puro hESCs was performed as previously described for other hESC lines [7], [8]. In brief, undifferentiated hESCs were dispersed to single cells with Versene and then plated in a high density monolayer in the presence of MEF-conditioned media supplemented with 5 ng/mL hbFGF. After confluence was reached, the cultures were induced to differentiate by switching to RPMI media (Gibco) containing 100 ng/mL recombinant activin A (R&D Systems), 1∶60 diluted Matrigel (BD), and insulin-free B27 supplement (Invitrogen). After 24 hours, the latter media was exchanged with insulin-free RPMI-B27 supplemented with 10 ng/mL recombinant BMP4 (R&D Systems). On days 5 and 7 post-induction with activin A, the cultures were re-fed with insulin-free RPMI-B27 without any exogenous growth factors and then this step was repeated using insulin-containing RPMI-B27 on day 9 and every other day thereafter.

### Transduction and Phenotyping of hESC-derived Cardiomyocytes

Differentiated cultures containing hESC-derived cardiomyocytes were dispersed with Versene and trypsin. A fraction of the cells were plated onto 0.1% PEI/0.5% gelatin coated chamber slides in RPMI-B27 medium supplemented with 10% FBS, 10 uM Y-27632 (Sigma), Polybrene (Millipore) and 5000 particles per cell of a lentivirus expressing Cre under the striated muscle-specific CK7 regulatory cassette, based on the muscle creatine kinase promoter (MCK) [9]–[11]. This medium was exchanged with RPMI-B27 at 24 hours after replating and transduction. Where indicated, cultures were subjected to selection with puromycin (1 µg/mL) at 48 hours following transduction with the MCK-CK7 lentiviral vector.

With each plating step, a separate fraction of dispersed cells was set aside for parallel analysis by flow cytomety. For this, cells were fixed in 4% paraformaldehyde and then labeled with either a mouse anti-cardiac troponin T (cTnT) antibody (1∶100 dilution, ThermoScientific, Cat# MS-295) or isotype control (1∶100, eBioscience, Cat# 14-4714). Detection was performed using an Alexafluor 660-conjugated anti-mouse secondary antibody (Molecular Probes). Flow cytometry and analysis was then performed as described above. Cells were gated against the isotype control as detected using a 633-nm argon laser plotting APC (660/20 filter).

### Immunofluorescence: Undifferentiated RUES2 mTmG-2a-Puro Cells

Cells were fixed for six minutes with 2% paraformaldehyde, permeabilized in PBS containing 0.025% Triton-X, and blocked in PBS containing 1.5% normal goat serum. Cells were stained with anti-Cre recombinase monoclonal antibody (1∶100, Covance, Cat#: MMS-106P) followed by secondary staining with Alexa Fluor 660 (1∶100, Molecular Probes). Nuclei were counterstained in PBS containing a 1∶2000 dilution of Hoechst 33342 (Thermo Scientific).

### Immunofluorescence: RUES2 mTmG-2a-Puro Cardiomyocytes

Cells were fixed with 2% paraformaldehyde and blocked with 1.5% normal goat serum. Cells were then stained with anti-α-actinin monoclonal (1∶100, Sigma, Cat#: A7811) and anti-GFP (1∶400, Molecular Probes, Cat#: A11122). Secondary staining was performed by incubation with species-specific Alexa Fluor conjugates (1∶500, Molecular Probes). Nuclei were counterstained in PBS containing a 1∶2000 dilution of Hoechst 33342 (Thermo Scientific).

## Results

Mazmudar et al. reported the creation of a non-selectable floxed, dual fluorescence “stoplight” reporter element (mTmG), in which the ubiquitously active CMV early enhancer/chicken beta-actin (CAG) promoter drives expression of either membrane-restricted tdTomato or eGFP (after Cre-mediated recombination) [3]. To facilitate the selection of cell populations not amenable to fluorescence-activated cell sorting, we modified the mTmG transgene to express puromycin N-acetyl transferase (i.e. puromycin resistance) as well as eGFP in cells that have undergone recombination. The resultant transgene is hereafter referred to as mTmG-2a-Puro. Next, to allow the selection of cells successfully modified with the mTmG-2a-Puro transgene, we also added a PGK-Neo cassette, cloned in the opposite orientation to the CAG-mTmG-2a-Puro cassette (**Figure 1**).

Standard methods for plasmid transfection are inefficient in hESCs [12], and successfully integrated transgenes are prone to silencing [13], making the generation of stable transfectant hESC lines very challenging. Lentiviral vectors are commonly used as an alternative in the hESC system [14], but they have limited packaging capacity, result in the random integration of an unknown number of gene copies, and are also prone to silencing. To avoid these limitations and because the mTmG-2a-Puro coding region exceeds the packaging capacity of most viral vectors anyway, we instead used zinc finger nuclease (ZFN) mediated recombination to insert the mTmG-2a-Puro construct into the human AAVS1 locus in RUES2 hESCs (**Figure 1A&B**). The AAVS1 “safe harbor” locus at 19q13.3 was chosen as the insertion target site, given reports that this locus has a relatively open chromatin configuration that facilitates strong, stable transgene expression in both undifferentiated and differentiated cell states [2], [15]. For this, we co-electroporated hESCs with two plasmids: one in which the floxed CAG-mTmG-2a-Puro reporter was flanked by homology arms and a second encoding containing a ZFN pair specific to the AAVS1 site [2]. The successfully targeted cells were purified with G418 selection, resulting in hESC cultures that were greater than 95% tdTomato^+^ cells by epifluorescent microscopy and flow cytometry (**Figure 2A&B**). We used Southern blot to confirm that selected transgenic hESC populations with strong, stable tdTomato expression had been properly targeted at the AAVS1 locus was confirmed by Southern blot (**Figure 2C**), and these cultures were shown to have a normal female karyotype (**Figure 2D**). Of note, following this protocol, we infrequently obtained tdTomato^+^ hESC populations that were later found by Southern blot to have resulted from random integration events. Interestingly, such cultures also showed highly variable tdTomato fluorescence (both from cell-to-cell and over time), and so they were discarded (**Figure S1**).

Next, we moved to studies to validate the functionality of the floxed stoplight reporter in the correctly targeted cultures, hereafter referred to as mTmG-2a-Puro hESCs. We began with studies in the pluripotent state, transducing undifferentiated transgenic hESCs with a lentiviral vector in which the ubiquitously expressed elongation factor 1α promoter drives Cre recombinase (hereafter, EF1α-Cre). After transduction, cells were analyzed for eGFP and tdTomato expression by flow cytometry at regular intervals (**Figure 3A&B**). eGFP expression was detected within 24 hours of EF1α-Cre transduction. By 48 hours post-transduction, distinct eGFP^+^/tdTomato^+^ and eGFP^−/^tdTomato^+^ cell populations had emerged. Between days 3 to 6 post-transduction, the eGFP^+^ population became increasingly distinct as the tdTomato fluorescence in these cells decreased. By day 6, ∼50% of the cells were either tdTomato^+^ or eGFP^+^ (**Figure 3B**), as expected given our transduction efficiency of ∼50% [**16**].

To demonstrate the capacity of our system to purify Cre-marked hESCs and their progeny, we next subjected the EF1α-Cre transduced cells to puromycin selection. After three days of selection, the resultant cell population was nearly 100% eGFP^+^ (**Figure 3C** **)**. Although some eGFP^+^ cells remained tdTomato^+^ at this time-point, rare tdTomato^+^/GFP^-^ cells were retained. Qualitatively similar results were obtained during fluorescence imaging of live cells treated with EF1α-Cre lentivirus (**Figure 3D**). When we immunostained the latter cultures with an antibody against Cre-recombinase, Cre expression was only detected in the nuclei of hESCs that also expressed eGFP (**Figure 3E**). Importantly, these data show that puromycin resistance is constrained in a cell autonomous manner to eGFP^+^ hESCs, greatly facilitating the purification of eGFP^+^ cells from an initially heterogeneous cell population.

Next, we validated the use of mTmG-2a-Puro reporter hESCs in the isolation of a specific cell type (in this case, cardiomyocytes) from a heterogeneous population of differentiated cells. For this, we differentiated mTmG-2a-Puro hESCs using a monolayer-based cardiac induction protocol, previously reported by our group [7], [8]. This protocol typically yields differentiated cultures in which a majority of the hESC progeny are cardiomyocytes. When we applied it to mTmG-2a-Puro hESCs, we obtained populations of 68±2.5% cardiac troponin T positive (cTnT^+^) cardiomyocytes (**Figure 4A&B**). To test the ability of genetic selection following cardiomyocyte-restricted Cre expression to yield an even higher degree of cardiac purity, we transduced these cultures with a lentiviral vector in which Cre expression is driven by a striated muscle-specific regulatory gene cassette (MCK-CK7), derived from modified portions of the mouse muscle creatine kinase gene enhancer and proximal promoter [10], [11]. We then dual-labeled the transduced cultures using antibodies against eGFP and the cardiomyocyte marker α-actinin. As expected, the cultures contained a mixture of recombined and non-recombined cardiomyocytes prior to puromycin selection (eGFP^+^ α-actinin^+^: 45.8±7.7%, eGFP^-^ α-actinin^+^: 34.5±2.9%, eGFP^+^ α-actinin^-^: 1.0±1.2%, eGFP^-^ α-actinin^-^: 19.5±8.7%) (**Figure 4C&D**). However, after puromycin selection, we obtained highly enriched populations of eGFP^+^ α-actinin^+^ cardiomyocytes (eGFP^+^ α-actinin^+^: 98.6±0.2%, eGFP^-^ α-actinin^+^: 0.8±0.2%, eGFP^+^ α-actinin^-^: 0.6±0.3%, eGFP^-^ α-actinin^-^: 0.0±0.0%) (**Figure 4C&D**). Taken collectively, these data show that, when combined with suitable vectors for cell-type specific expression of Cre recombinase, the mTmG-2a-Puro floxed reporter hESC line described here can be used to isolate specific cell populations that approach homogeneity.

## Discussion

Genetic approaches to fate mapping and the selective enrichment of specific cell types have provided useful insights into the developmental potential of stem and progenitor cells in vivo and in vitro [17]. However, our ability to apply these approaches and isolate specific subpopulations has been severely limited in the hESC system. In this report, we describe the generation and validation of mTmG-2a-Puro RUES2 hESCs, which contain a unique floxed reporter transgene that includes both fluorescent reporters and an antibiotic resistance cassette. We also show that this stably integrated transgene allows the labeling and selection of Cre-recombined cells in both undifferentiated and differentiated cultures in a promoter-specific manner. In this study, we have validated the mTmG-2a-Puro concept using the RUES2 hESC line, but the same approach and genetic constructs should be applicable to any human cells, including other hESC lines and human induced pluripotent stem cell (iPSCs). For example, insertion of the mTmG-2a-Puro transgene into patient-derived iPSC lines might be particularly useful for future disease modeling or drug screening efforts that require the isolation of a particular differentiated cell type (e.g. cardiomyocytes).

In a previous study, Nolden et al. described a floxed two-color reporter hESC line as part of a study designed to test the activity of cell-permeant Cre-recombinase [18]. Here, we have further refined the floxed “stoplight” reporter concept, while also developing genetic tools that could be used to conveniently insert such a reporter into multiple human pluripotent stem cell lines. Indeed, our ZFN-based strategy offers a number of advantages over conventional plasmid transfection or viral vectors: all of the successfully targeted cells should have a single copy of the reporter transgene in a defined genetic locus, plus it can accommodate larger, more complicated constructs such as mTmG-2a-Puro. The mTmG-2a-Puro transgene incorporates a number of features that we expect will be useful for investigators in the field. First, it encodes for two bright fluorescent proteins, both of which also happen to be membrane-restricted. (This subcellular localization aids in distinguishing true fluorescent signal from autofluorescent background, and it is particularly helpful in recognizing structures such as axonal projections [3].) Second, we have added a Cre-dependent puromycin selection component, which can be used purify a single cell type from a heterogeneous starting population. We predict that this feature will prove particularly useful in the isolation of differentiating cell types that may be otherwise distinguished only by a weak or transiently activated promoter.

Finally, it should be noted that the mTmG-2a-Puro RUES2 hESCs described in this study do not represent a clonal hESC line, so we cannot exclude a minority population of randomly integrated cells (albeit below the level of detection by Southern blot). If a particular application required a 100% homogenous transgenic hESC population, this limitation could be overcome by dilution cloning and expansion from a single cell. While time-consuming and technically challenging, such methodologies have been successfully applied to hESCs [19]. In practice, we have found that the polyclonal mTmG-2a-Puro RUES2 hESC populations that result from G418 selection exhibit very little phenotypic variation and so should be suitable for most fate-mapping and cell purification applications.

## Supporting Information

Figure S1 **Multiple transgene integrations result in variable phenotypes.** ZFN transgene integration occasionally yields cells with multiple, random transgene integrations. (**A**) Diagram of basic mTmG-2a transgene where any gene can be inserted after the 2a sequence. Diagram indicates NheI, NdeI and KpnI restriction sites for Southern blot assay. The orange bar represents the binding site for the non-locus-specific Southern blot Neomycin probe. AAVS1 targeted transgenes will have a band at 3.2kb by Southern blot. (**B**) Southern blot from three different mTmG-2a populations and wild type hESCs. Only population 2 shows a correctly targeted transgene at the AAVS1 locus, while populations 1, 2 and 3 all show multiple random integrations. (**C**) Phase contrast and tdTomato fluorescence photomicrographs of populations 1, 2, 3 and mTmG-2a-Puro cells showing heterogeneous tdTomato fluorescence intensity in the populations with multiple transgene insertions and homogeneous tdTomato expression in the properly targeted, mTmG-2a-Puro cells.
(TIF)
