# Supplementary figures and images for: Targeted Genomic Integration of a Selectable Floxed Dual Fluorescence Reporter in Human Embryonic Stem Cells
Source: PLoS One. 2012 Oct 10;7(10):e46971. doi: 10.1371/journal.pone.0046971 (PMC3468579; doi:10.1371/journal.pone.0046971)

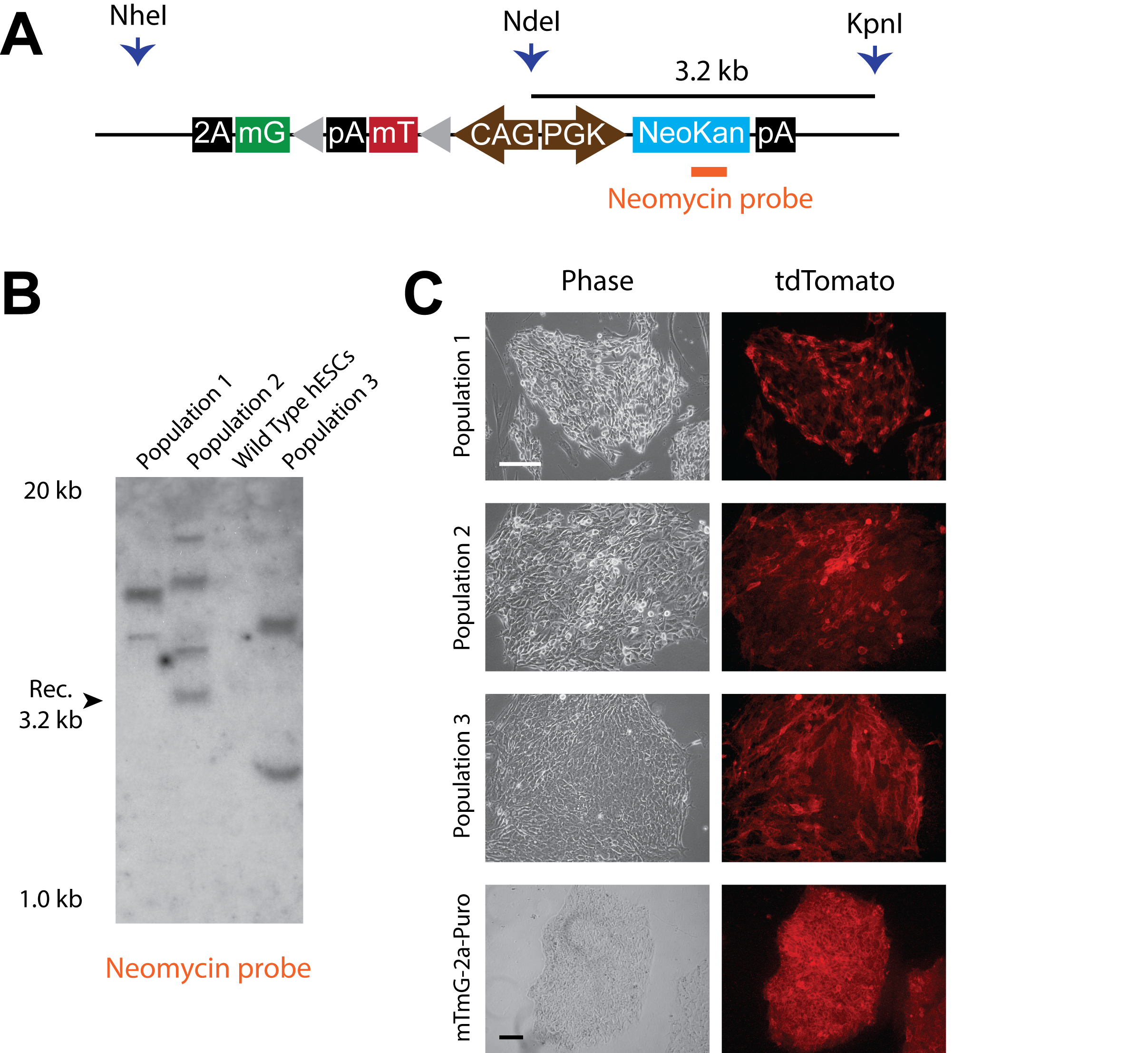

Supplement: Figure S1 — Multiple transgene integrations result in variable phenotypes. ZFN transgene integration occasionally yields cells with multiple, random transgene integrations. (A) Diagram of basic mTmG-2a transgene where any gene can be inserted after the 2a sequence. Diagram indicates NheI, NdeI and KpnI restriction sites for Southern blot assay. The orange bar represents the binding site for the non-locus-specific Southern blot Neomycin probe. AAVS1 targeted transgenes will have a band at 3.2kb by Southern blot. (B) Southern blot from three different mTmG-2a populations and wild type hESCs. Only population 2 shows a correctly targeted transgene at the AAVS1 locus, while populations 1, 2 and 3 all show multiple random integrations. (C) Phase contrast and tdTomato fluorescence photomicrographs of populations 1, 2, 3 and mTmG-2a-Puro cells showing heterogeneous tdTomato fluorescence intensity in the populations with multiple transgene insertions and homogeneous tdTomato expression in the properly targeted, mTmG-2a-Puro cells. (TIF) [file pone.0046971.s001.tif]
